# HOW ARE MULTIPLE CHRONIC CONDITION COMBINATIONS DIFFERENTIALLY ASSOCIATED WITH HEALTH SERVICE USE?

**DOI:** 10.1093/geroni/igac059.558

**Published:** 2022-12-20

**Authors:** Aaron Ogletree, Benjamin Katz

**Affiliations:** National Institutes of Health, Washington, District of Columbia, United States; Virginia Tech, Blacksburg, Virginia, United States

## Abstract

A growing body of literature describes important advances in the study of chronic conditions, most notably a paradigm shift from the study of individual chronic conditions to the study of multiple chronic conditions (MCCs). Despite these advances, little research has explored MCC combinations, and almost no published research has explored how MCC combinations are related to health service utilization. Using data from the 2018 wave of the Health and Retirement Study, we categorized 16,447 older adults into one of 32 groups using self-reports of five prevalent chronic conditions: arthritis, diabetes, heart problems, hypertension, and respiratory problems. ANOVAs assessed associations between MCC combinations and two self-report measures of health service use: (1)number of medical visits, including emergency room visits, clinic visits, and house calls; and (2)total out-of-pocket costs for major medical expenses. Results show that older adults with more conditions had a greater number of medical visits (p< 0.0001). The pattern between total number of conditions and out-of-pocket costs is less clear, though still varied significantly (p< 0.01). Findings demonstrate variability in outcomes among MCC combinations with the same total number of conditions. Those in the Heart-Hypertension-Respiratory group averaged 9 medical visits while those in the Arthritis-Diabetes-Heart group averaged 16. Adults in the Diabetes-Heart group averaged $2,546 in out-of-pocket costs, which is nearly double the costs reported by the Diabetes-Hypertension group ($1,254). Findings highlight complex associations of MCC combinations with health service use, and can inform resource allocation, policy priorities, and care planning among providers serving older adults with MCCs.

